# Signal peptide variation in cyst lectins as a potential marker for pathogenic *Acanthamoeba* spp.

**DOI:** 10.1186/s13071-026-07359-4

**Published:** 2026-03-16

**Authors:** Chih-Ming Tsai, Wei-Hung Cheng, Wei-Chen Lin

**Affiliations:** 1 Department of Parasitology, College of Medicine, National Cheng Kung University, Tainan City, Taiwan; 2 Institute of Basic Medical Sciences, College of Medicine, National Cheng Kung University, Tainan City, Taiwan

## Abstract

**Background:**

*Acanthamoeba* species are free-living protists widely distributed in natural and artificial environments, including freshwater, soil, and water-associated facilities. Some isolates can cause opportunistic human infections, such as *Acanthamoeba* keratitis and granulomatous amoebic encephalitis. The life cycle of *Acanthamoeba* includes an active trophozoite stage and a dormant cyst stage. The cyst is surrounded by a complex double-layered wall composed of cellulose and multiple structural proteins, including cyst wall lectins. While the architecture and molecular composition of the cyst wall have been investigated, inter-isolate sequence variation of cyst wall components remains poorly characterized.

**Methods:**

We performed a genome-based comparative analysis of 31 previously characterized cyst wall lectins, analyzing the homologous sequence of each lectin across 31 publicly available *Acanthamoeba* genomes. Isolates were classified as clinical or environmental on the basis of their reported sources. Sequence similarity-based clustering was conducted to identify lectins associated with clinical isolates. For selected genes, sequence features at the 5′ region were further examined and experimentally validated by polymerase chain reaction using long-term axenic clinical isolates maintained at National Cheng Kung University Hospital.

**Results:**

Similarity-based clustering identified eight cyst wall lectins that formed clusters enriched for clinical isolates. Among these, three lectins showed pronounced sequence variation at the 5′ region in multiple clinical isolate genomes. Polymerase chain reaction (PCR) validation confirmed that these variations were present in clinical isolates and were not attributable to genome assembly artifacts. Sequence analysis suggested that these variations may affect signal peptide features at the amino terminus of the proteins.

**Conclusions:**

Our findings reveal previously underexplored sequence diversity in cyst wall lectins across *Acanthamoeba* isolates. This diversity may contribute to differences in cyst wall architecture between clinical and environmental isolates. These results provide new insight into cyst-associated molecular features that may be linked to pathogenic potential and offer a foundation for future studies on cyst-mediated adaptation and infection.

**Graphical abstract:**

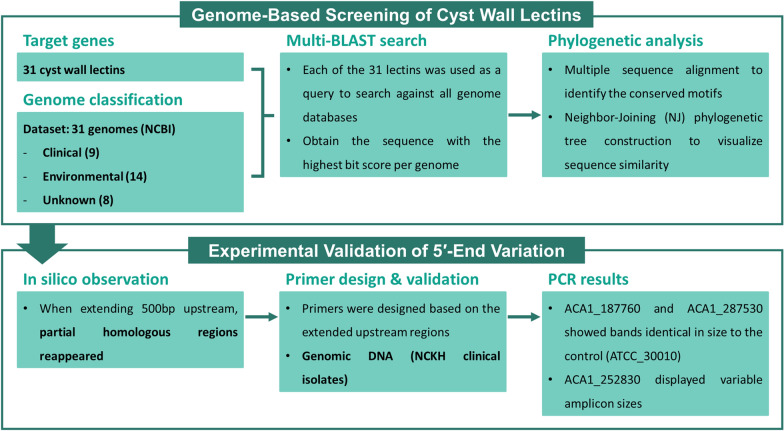

## Background

*Acanthamoeba* spp. are free-living protozoa that can cause opportunistic infections in humans. They cause sight-threatening *Acanthamoeba* keratitis (AK) and granulomatous amoebic encephalitis, a rare but frequently fatal disease in immunocompromised individuals [1–3]. In recent decades, the global incidence of *Acanthamoeba* infections has increased and is considered to be associated with the widespread use of contact lenses [4, 5]. Consequently, *Acanthamoeba* keratitis continues to occur even in regions with advanced public health infrastructure [6, 7].

The life cycle of *Acanthamoeba* comprises two stages: an active trophozoite and a dormant cyst. Trophozoites feed on surrounding microorganisms through phagocytosis, while host cell damage is primarily mediated by secreted proteases and pore-forming proteins, and partly by trogocytosis. Some studies have suggested that this interaction may enhance the virulence of coexisting microbes [8–10]. By contrast, the cyst form is characterized by a double-layered cellulose wall that confers resistance to a wide range of environmental stresses, including nutrient deprivation, osmotic fluctuations, extreme temperatures, pH shifts, and exposure to antimicrobial agents [11–13]. Upon exposure to harsh environmental conditions, the trophozoite undergoes encystation and transforms into the cyst stage. This high resilience underlies the ubiquity of *Acanthamoeba* in diverse habitats such as soil, freshwater, seawater, and even man-made environments [14]. The cyst’s resistance to most currently available contact lens solutions and anti-amoebic drugs often leads to treatment failure and infection recurrence [13, 15, 16].

As a result, human exposure to *Acanthamoeba* is frequent. Previous studies indicated that anti-*Acanthamoeba* IgA antibodies are commonly detected in the general population [17]. However, while exposure to *Acanthamoeba* is common, the development of symptomatic disease is relatively rare, suggesting that successful invasion requires specific conditions. Therefore, elucidating these pathogenic mechanisms remains a key area of research. During the infection process, *Acanthamoeba* must transition through multiple environments, ranging from natural reservoirs to human-associated materials and host tissues, such as the cornea [18, 19]. Although cysts themselves do not directly cause host cell damage, the ability to balance encystation and excystation is critical for survival and successful infection [20]. Failure to encyst promptly can lead to death, whereas excessive encystation or delayed excystation impedes population growth and host colonization. However, the relationship between cyst physiology and pathogenicity remains poorly understood.

Currently, *Acanthamoeba* spp. lack definitive molecular markers that reliably differentiate pathogenic from nonpathogenic lineages. Although clinical isolates are confirmed to exhibit pathogenic potential, environmental and unclassified isolates cannot be assumed to be nonpathogenic, as many environmental isolates have been implicated in keratitis and other infections. This uncertainty necessitates the use of operational definitions when comparing genomic features associated with pathogenicity. However, we hypothesize that specific genetic features in clinical isolates may underlie the pathogenic potential of these amoebae. In this study, *Acanthamoeba* isolates were categorized into clinical and nonclinical isolates, under the working hypothesis that traits commonly shared among clinical isolates represent putative determinants of pathogenicity.

Our analysis focused on cyst wall lectins, which form major structural components of the *Acanthamoeba* cyst wall together with cellulose [21]. These lectins fall into three families, all of which bind strongly to cellulose. The Luke lectins contain two CBM49 carbohydrate-binding modules, outline the small, flat ostioles in the single-layered primordial wall, and localize to the endocyst layer and ostioles of mature cyst walls. The Leo lectins contain two unique domains, each with eight cysteine residues, and localize to the endocyst layer and ostioles. The Jonah lectins contain a single choice-of-anchor A (CAA) domain, are produced early during encystation, and localize to the ectocyst layer. Their distinct spatial distributions and stage-specific expression patterns indicate that encystation is a highly ordered and complex process [21, 22]. Cyst wall lectins have also been implicated in the formation of ostioles, specialized structures connecting the inner and outer walls, which are thought to play roles in environmental sensing and excystation. Consequently, cyst wall lectins are likely involved not only in cyst wall formation but also in adaptive responses to environmental cues; however, this relationship remains largely unexplored.

**Fig. 1:**
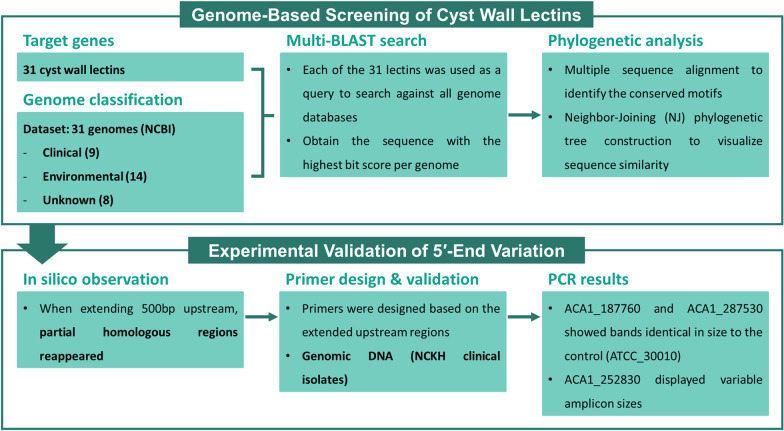
Overview of the analytical workflow used to identify and validate 5′-end variation in cyst wall lectins. The study consisted of two major components: genome-based screening (top) and experimental validation (bottom). In the genome-based screening, 31 cyst wall lectins were selected as target genes and classified across 31 publicly available *Acanthamoeba* genomes (clinical, environmental, and unclassified). Each lectin was used as a query in a multi-BLAST search to retrieve the highest-scoring homologous sequence from each genome. Retrieved sequences were subjected to multiple sequence alignment and neighbor-joining sequence similarity analysis to identify conserved motifs and potential clinically associated clusters. In the experimental validation, in silico extension of 500 base pairs (bp) upstream of selected genes revealed partially homologous regions in clinical isolates. Primers were designed on the basis of these extended upstream sequences and tested using genomic DNA from NCKH clinical isolates

To further investigate the molecular features potentially associated with pathogenicity, we conducted sequence similarity analysis of cyst lectins across diverse *Acanthamoeba* species. An overview of the computational and experimental workflow used in this study is summarized in Fig. 1. Among the 31 known lectin genes [21], 8 were found to be associated with clinical isolates. Comparative analyses of gene structures revealed that three of these genes exhibited sequence gaps within the signal peptide regions of the clinical variants. Re-examination of the corresponding genomic sequences indicated that these gaps resulted from high sequence variability at the 5′ ends. We subsequently verified the 5′ sequence diversity in clinical isolates previously collected from National Cheng Kung University Hospital [23] and confirmed that some cyst wall lectins lacked signal peptides through bioinformatic predictions. Although the functional consequences of these alterations remain unclear, our findings suggest that structural diversity among cyst wall lectins may contribute to variations in cyst morphology and could be linked to the pathogenic potential of different *Acanthamoeba* isolates.

## Methods

### Culture of *Acanthamoeba*

*Acanthamoeba* was cultured as described previously [2]. Briefly, *A. castellanii* was maintained in proteose peptone–yeast extract–glucose (PYG) medium (pH 6.5) at 28 °C in culture flasks. Trophozoites were harvested after 3–5 days of cultivation during the logarithmic growth phase. The American Type Culture Collection (ATCC) reference strains used in this study were *A. castellanii* ATCC 30010, ATCC 50492, and ATCC 50370. ATCC_30010 (Neff strain) is a soil-derived isolate commonly used as a representative nonclinical laboratory strain. ATCC_50370 and ATCC_50492 were isolated from patients with AK and are widely used as clinical reference strains. These strains were selected to provide well-characterized environmental and clinical references for comparative analysis [23–27]. Clinical isolates (NCKH_A, NCKH_B, NCKH_C, NCKH_D, NCKH_G, and NCKH_H) were previously isolated from corneal ulcer specimens of patients diagnosed with AK at National Cheng Kung University Hospital (NCKUH), Tainan, Taiwan, and had been maintained as long-term axenic cultures before this study [23, 28]. No new patient samples were collected for the present work. The use of patient-derived materials was reviewed and approved by the Institutional Review Board of NCKUH.

### DNA extraction

A total DNA Mini Kit (QIAGEN, Germany) was used to extract DNA from the pellets. Total DNA was stored at −20 ℃. The concentration and A260/2A80 ratio of the DNA were measured with an ND-1000 (NanoDrop, Thermo Fisher Scientific, USA).

### Polymerase chain reaction (PCR)

For the detection and sequencing of the genomic DNA sequences of the cyst lectins in *Acanthamoeba* clinical isolates, PCR was applied. Each round of PCR was carried out in a 20.0 μL reaction, comprising 1.0 μL of DNA template, 1.0 μl of 10 μM for each primer, 10.0 μL of Taq 2× Master Mix Red (Ampliqon, Denmark), and ddH_2_O. The PCR conditions included initial denaturation at 95 °C for 1 min, followed by 35 cycles of 15 s at 95 °C, 15 s at 55 °C, and 30 s at 72 °C, concluding with a final extension step at 72 °C for 7 min.

Specific primers were designed to amplify the genomic DNA fragments encoding cyst lectins from *Acanthamoeba* clinical isolates. The primer sequences, target genes, and expected product sizes are summarized in Table 1. Product sizes were determined on the basis of the reference genome sequence of *Acanthamoeba* *castellanii* strain ATCC_30010.

**Table 1:** Primers used for the amplification of cyst lectin genes in *Acanthamoeba* clinical isolates

Target gene	Forward (5′ → 3′)	Reverse (5′ → 3′)	Product size (bp)	Reference strain
ACA1_187760	GAAGCGTTTCATTCTACTCGCC	TGCCGACTCCCAGATGTAGC	398	ATCC_30010
ACA1_252830	ATGAAGAGCTCCTTCTTGACACT	TCTGGTAGGTGTAGGTGGCA	681	ATCC_30010
ACA1_287530	TGAAGCAATTCCTCCTCCTCGC	GGACTCCCAGATGTAGCCGA	382	ATCC_30010

### Comparative genomic analysis

Comparative genomic analyses were conducted to identify homologous sequences of target genes among *Acanthamoeba* species. Genome assemblies of 30 *Acanthamoeba* isolates, excluding the reference strain ATCC_30010, were downloaded from the National Center for Biotechnology Information (NCBI) Genome database. Each genome was formatted into a local nucleotide BLAST database using makeblastdb to enable subsequent searches.

The nucleotide sequences of the target genes from *A. castellanii* ATCC_30010 were used as queries for BLASTN searches against all local *Acanthamoeba* databases. The analyses were automated using in-house Python scripts that processed multiple query sequences sequentially and performed alignments against each genome database. For each query, both the forward and reverse strands of the genomic sequences were considered during the alignment process.

The highest-scoring hits (based on bit score) for each query were automatically extracted from the BLAST outputs, and the corresponding genomic regions were retrieved. When the aligned regions were located on the complementary strand, the sequences were reverse-complemented to ensure correct orientation. Each retrieved sequence was labeled according to the genome’s metadata as clinical or environmental on the basis of the NCBI assembly information (genome_labels.txt), facilitating subsequent comparative analyses between strains of different origins.

### Genome classification and target gene selection

A total of 31 *Acanthamoeba* genomes were retrieved from the NCBI GenBank database for comparative analysis (Table 2). On the basis of metadata annotations, the genomes were categorized into clinical (*n* = 9), environmental (*n* = 14), and unclassified (*n* = 8) groups. This classification was used as the reference framework for subsequent analyses to evaluate the relationship between genomic background and cyst wall lectin diversity.

**Table 2:** The information on 31 *Acanthamoeba* spp. genomes

Assembly	Organism name	Strain	Whole-genome sequencing (WGS) project	Category
GCA_046056075.1	*Acanthamoeba griffini*	Sawyer	JBJUIM01	Clinical
GCA_000826365.1	*Acanthamoeba royreba*		CDEZ01	Clinical
GCA_000826385.1	*Acanthamoeba rhysodes*		CDFC01	Clinical
GCA_046056115.1	*Acanthamoeba castellanii*	1BU	JBJUIN01	Clinical
GCA_000826285.1	*Acanthamoeba lenticulata*		CDFG01	Clinical
GCA_002105255.1	*Acanthamoeba lenticulata*	Feb-72	MSTW01	Clinical
GCA_027943245.1	*Acanthamoeba sp. SK_2022b*		JANDJZ01	Clinical
GCA_027943295.1	*Acanthamoeba sp. SK_2022a*		JANEZP01	Clinical
GCA_000826485.1	*Acanthamoeba castellanii*		CDFL01	Clinical
GCA_000313135.1	*Acanthamoeba castellanii*	Neff	AHJI01	Environmental
GCA_002179805.1	*Acanthamoeba lenticulata*	PT-14	NAVB01	Environmental
GCA_000826245.1	*Acanthamoeba astronyxis*		CDFH01	Environmental
GCA_046055985.1	*Acanthamoeba terricola*	Neff	JBJUIL01	Environmental
GCA_000826305.1	*Acanthamoeba healyi*		CDFA01	Environmental
GCA_002025285.2	*Acanthamoeba comandoni*	Pb30/40	MRZZ02	Environmental
GCA_021020595.1	*Acanthamoeba castellanii*	C3	JAJGAO01	Environmental
GCA_021020605.1	*Acanthamoeba castellanii*	Neff	JAJGAP01	Environmental
GCA_000193105.1	*Acanthamoeba castellanii*	Neff	AEYA01	Environmental
GCA_903821525.1	*Acanthamoeba castellanii*	Namur	CAIJLO01	Environmental
GCA_000826345.1	*Acanthamoeba polyphaga*		CDFK01	Environmental
GCA_027944975.1	*Acanthamoeba sp. SK_2022c*		JANDKB01	Environmental
GCA_937900865.1	*Acanthamoeba castellanii*		CALCFY01	Environmental
GCA_000826325.1	*Acanthamoeba palestinensis*		CDFD01	Environmental
GCA_902749335.1	*Acanthamoeba triangularis*	SH 621	CACVKS01	Unsure
GCA_000826265.1	*Acanthamoeba culbertsoni*		CDFF01	Unsure
GCA_000826405.1	*Acanthamoeba divionensis*		CDFI01	Unsure
GCA_001567625.1	*Acanthamoeba polyphaga*	Linc Ap-1	LQHA01	Unsure
GCA_000826445.1	*Acanthamoeba quina*		CDFN01	Unsure
GCA_000826465.1	*Acanthamoeba mauritaniensis*		CDFE01	Unsure
GCA_000826425.1	*Acanthamoeba lugdunensis*		CDFB01	Unsure
GCA_000826505.1	*Acanthamoeba pearcei*		CDFJ01	Unsure

The 31 cyst wall lectin genes characterized by Magistrado et al., (12 Luke, 14 Leo, and 5 Jonah) were selected as target sequences. Each lectin gene was used as a query to search against all genome assemblies using BLAST, and the sequence with the highest bit score per genome was extracted for downstream alignment and sequence similarity analysis. Multiple sequence alignment and neighbor-joining (NJ) tree construction were performed to visualize sequence similarities and to identify potential clinical branches, defined as similarity-based clusters containing five or more clinical isolates.

### Sequence similarity analysis

Sequence similarity analysis was performed individually for each of the 31 gene sequences obtained from comparative genomic analysis. The nucleotide sequences corresponding to each gene were aligned using MEGA version 12 with the default multiple sequence alignment parameters. Similarity-based clustering trees were subsequently constructed using the neighbor-joining (NJ) method implemented in MEGA, with pairwise deletion for gaps and bootstrap analysis based on 1000 replicates to assess the robustness of the inferred clades. The resulting similarity-based clustering trees were exported and visualized using Jalview, which was employed to improve the clarity of branch structures, color coding, and sequence labeling for comparative interpretation across *Acanthamoeba* isolates.

### Prediction of signal peptide

The 5′ terminal sequences obtained from PCR and sequencing of clinical *Acanthamoeba* isolates were reassembled to reconstruct the complete coding regions of the target genes. The assembled nucleotide sequences were analyzed using FGENESH (SoftBerry Inc.) to predict exon–intron structures on the basis of eukaryotic gene models. The predicted messenger RNA (mRNA) sequences were subsequently translated into amino acid sequences using the Expasy Translate Tool (https://web.expasy.org/translate/). The resulting protein sequences were analyzed with SignalP 6.0 to predict the presence or absence of N-terminal signal peptides. Sequences predicted to contain a signal peptide were further examined for cleavage site positions and the overall likelihood scores provided by SignalP to confirm the presence of secretion-associated motifs.

## Results

### Sequence similarity analysis identified clinical clusters of cyst wall lectins

Because no definitive molecular markers currently exist to distinguish pathogenic and nonpathogenic *Acanthamoeba*, clinical isolates were used as the only group with confirmed pathogenic potential. In this dataset, nine genomes were classified as clinical isolates, and clusters containing at least five clinical isolates were treated as clinical-majority clusters. This operational definition was applied to avoid overinterpreting sporadic clinical sequences and to ensure that the identified clades represent patterns dominated by clinical genomes rather than incidental grouping.

**Fig. 2:**
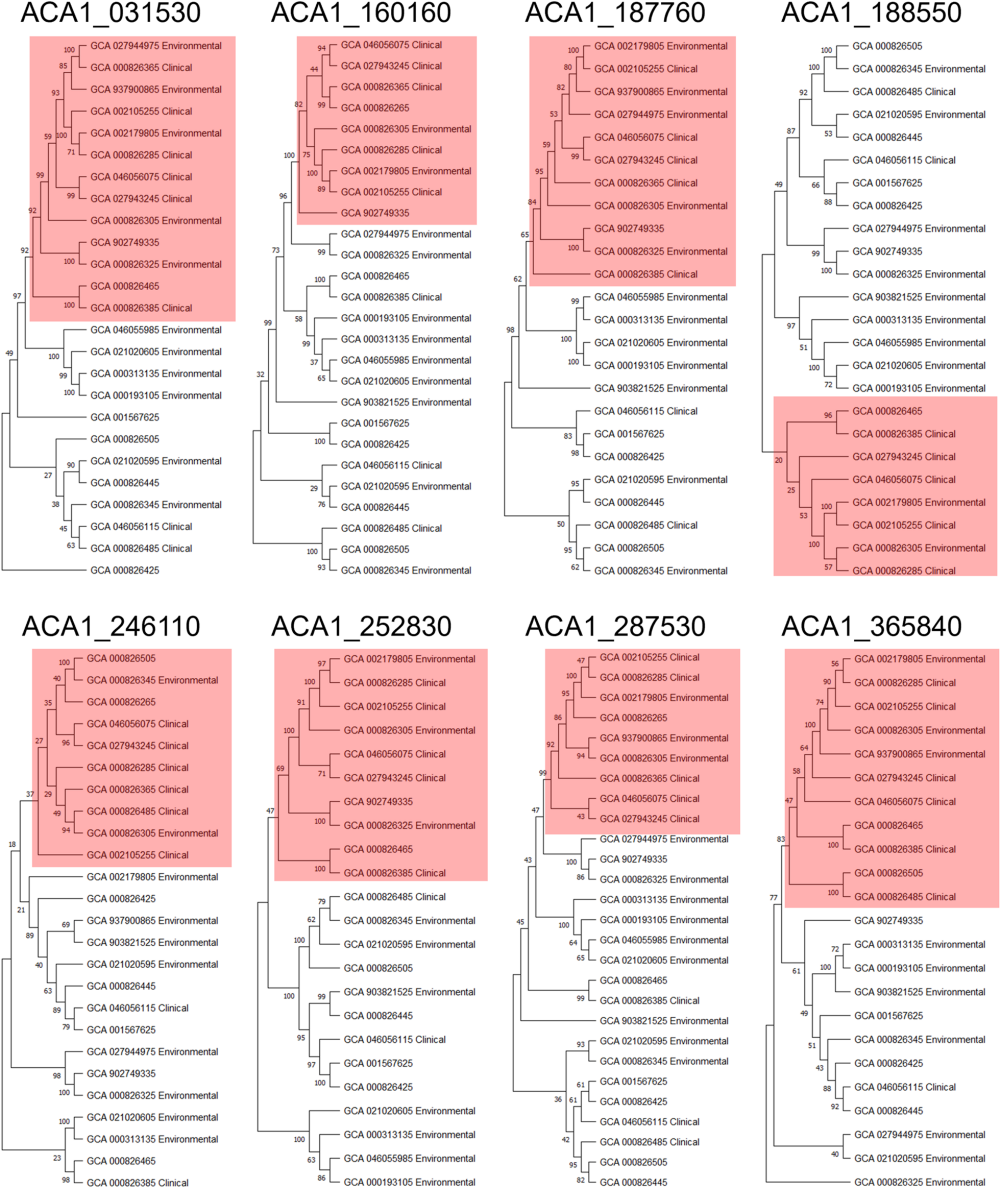
Similarity-based clustering trees of eight cyst wall lectins showing clinical clusters. Neighbor-joining similarity-based clustering trees were constructed for the eight lectin genes identified as forming clinical clusters: ACA1_031530, ACA1_160160, ACA1_187760, ACA1_188550, ACA1_246110, ACA1_252830, ACA1_287530, and ACA1_365840. For each gene, homologous sequences retrieved from 31 publicly available *Acanthamoeba* genomes were aligned and used to infer sequence similarity. Branches shaded in red represent clusters in which five or more clinical isolates were grouped together, meeting the predefined criterion for clinical-majority clusters. Both clinical and nonclinical genomes may be present within these clusters, reflecting the current limitations in distinguishing pathogenic potential solely on the basis of genome annotations. Bootstrap values (1000 replicates) are shown at key nodes

Similarity-based clustering trees generated from the 31 cyst wall lectins revealed eight genes that formed clinically associated clusters (Fig. 2). These clusters were enriched for clinical isolates, although environmental and unclassified genomes were also present, indicating that the grouping is not exclusive but reflects a clinically dominant pattern. Among these lectins, six belonged to the Luke family, including ACA1_031530, ACA1_160160, ACA1_187760, ACA1_246110, ACA1_252830, and ACA1_287530. The remaining two genes, ACA1_188550 and ACA1_365840, belonged to the Leo family. All eight lectins are classified as inner wall lectins, which suggests that genes associated with inner cyst wall formation exhibit lineage patterns linked to clinical isolates.

### Sequence characteristics of clinical cluster genes at the 5′ region

**Fig. 3:**
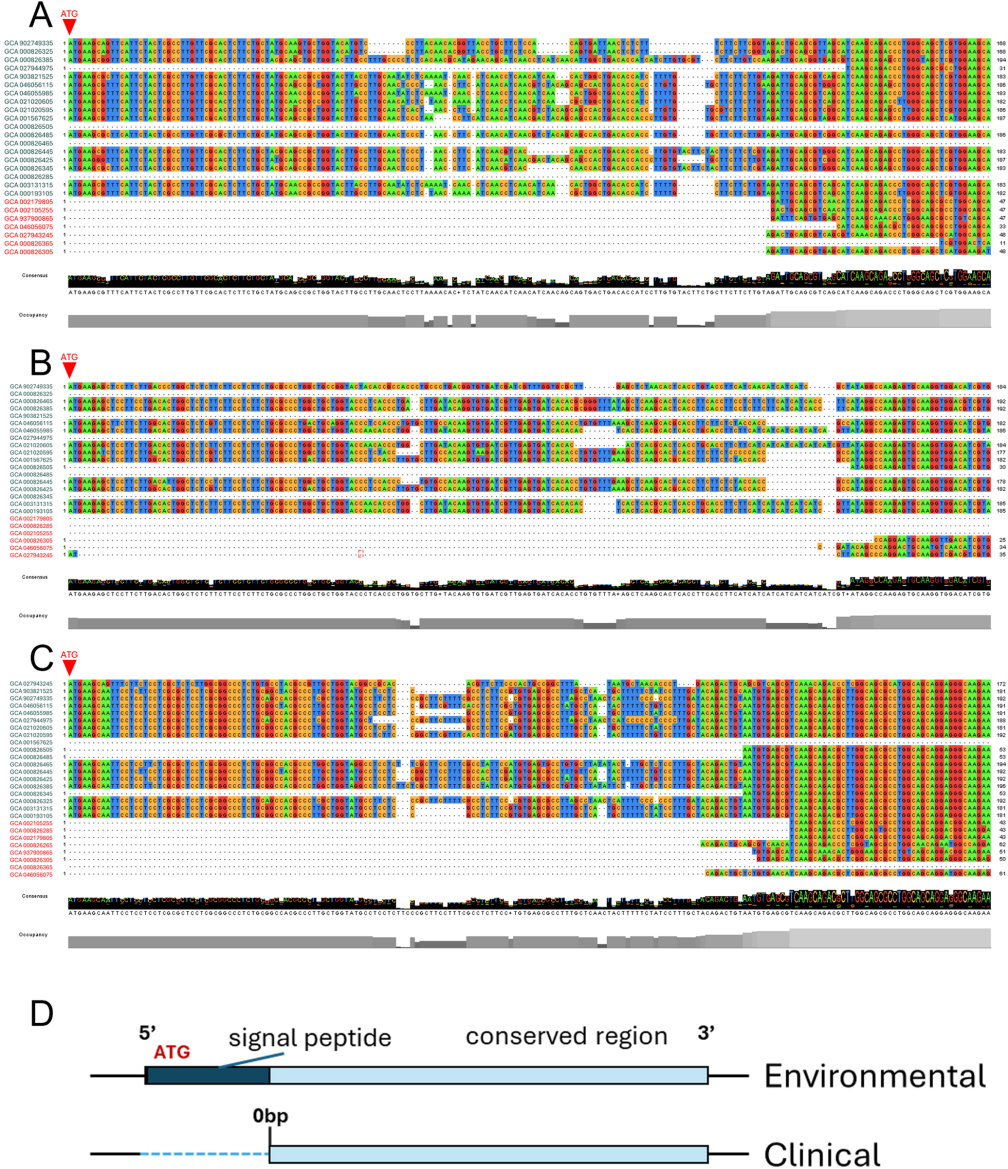
A 5′-end sequence variation of three lectins associated with clinical-majority clusters. **A–C** Multiple sequence alignments of ACA1_187760 (**A**), ACA1_252830 (**B**), and ACA1_287530 (**C**) constructed using genomic sequences from 31 *Acanthamoeba* isolates. Environmental isolates are shown in green, and clinical isolates are highlighted in red. All three lectins exhibited a prominent 5′-end gap among several clinical isolates, whereas environmental isolates displayed contiguous sequence coverage in the same region. The alignment reveals a clear boundary between the variable 5′-terminal region and the downstream conserved domain shared across isolates. **D** Schematic illustration summarizing the observed difference between environmental and clinical genomes at the 5′ region

Among the eight lectins that formed clinical clusters, three genes showed a clear and recurring feature in the multiple sequence alignment. These genes, ACA1_187760, ACA1_252830, and ACA1_287530, consistently showed a prominent 5′-end gap among several clinical isolates (Fig. 3). All cyst wall lectins contain a predicted signal peptide near the annotated 5′ region, which corresponds to the N-terminal secretion motif required for localization to the cyst wall. However, the presence of a 5′-end gap in the three genes raised the possibility that the annotated N-terminal region might be incomplete in clinical genomes.

**Fig. 4:**
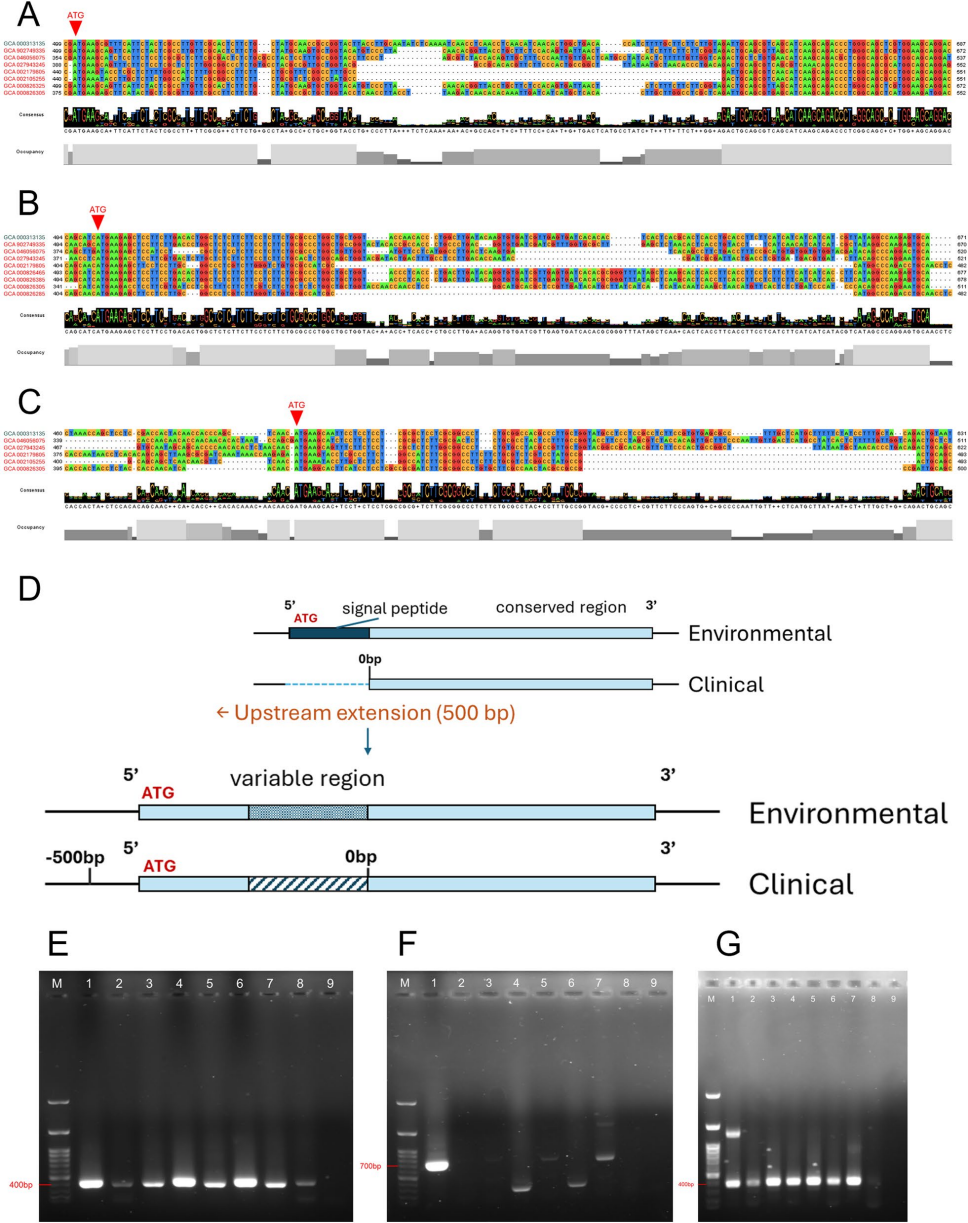
Experimental validation of 5′-end variation in clinical *Acanthamoeba* isolates. **A–C** Multiple sequence alignments of ACA1_187760 (**A**), ACA1_252830 (**B**), and ACA1_287530 (**c**) showing that several clinical isolates (highlighted in red) lack contiguous sequence coverage at the 5′ region, whereas environmental isolates retain a complete 5′ segment including the predicted start codon (ATG). This gap represents the most prominent sequence difference observed among clinical isolates for these three lectins. **D** Schematic representation of the comparative 5′-end structures. The upper panel illustrates the absence of the upstream region and signal peptide-encoding segment in several clinical genomes. Extending the clinical genomes 500 bp upstream enabled partial recovery of homologous sequence regions. The lower panel shows the reconstructed upstream architecture, indicating a variable 5′ region in clinical isolates compared with the conserved arrangement in environmental genomes. **E–G** PCR validation of upstream sequence restoration using genomic DNA from nine clinical isolates (lanes 1–9). **E** ACA1_187760, **F** ACA1_252830, and **G** ACA1_287530

To investigate this possibility, genomic sequences extending 500 base pairs upstream of the annotated start site were retrieved. Extension of the upstream regions partially restored homologous segments in several clinical isolates, and a putative start codon was identifiable within the extended sequences. These observations show that additional upstream content exists beyond the annotated boundaries in multiple genomes (Fig. 4a–d).

Primers were subsequently designed on the basis of these extended upstream sequences. Using genomic DNA from clinical isolates collected at NCKH, PCR amplification was performed for the three genes. ACA1_187760 and ACA1_287530 produced amplicons that were similar in size to the control strain ATCC_30010, whereas ACA1_252830 yielded amplicons of variable length across different clinical isolates (Fig. 4e–g).

### Signal peptide prediction reveals 5′-end sequence diversity among clinical isolates

**Fig. 5:**
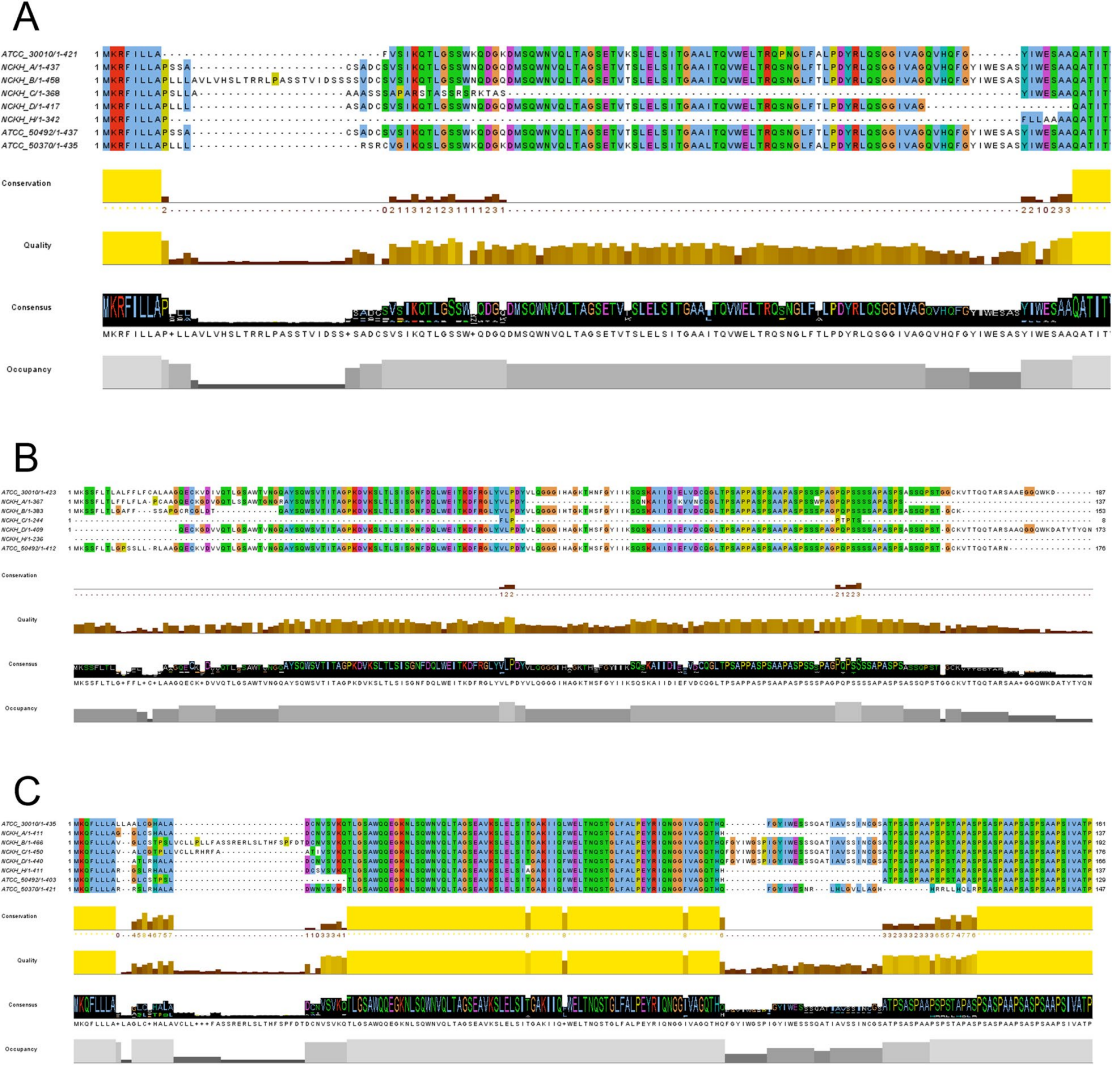
Predicted amino-acid sequence alignment of the reconstructed 5′ regions of three clinical-cluster lectins. **A** ACA1_187760, **B** ACA1_252830, and **C** ACA1_287530. For each gene, PCR-amplified 5′-terminal sequences from *Acanthamoeba* isolates were assembled to reconstruct full-length coding regions. The resulting amino-acid sequences were aligned to evaluate variation in the N-terminal region, including the predicted signal peptide. Sequence conservation, quality, consensus, and occupancy plots (Jalview outputs) highlight regions of divergence across isolates. These alignments correspond to the SignalP 6.0 predictions summarized in Table 1 and illustrate the isolate-specific differences in the N-terminal features of lectins associated with clinical-majority clusters

The PCR products obtained from clinical isolates were reassembled to restore the 5′-terminal sequences. The assembled nucleotide sequences were converted into predicted coding sequences on the basis of exon–intron structures generated by FGENESH. The resulting mRNA sequences were translated into amino acid sequences and were subjected to signal peptide prediction using SignalP 6.0. The resulting amino acid alignments demonstrated distinct N-terminal sequence variability among isolates (Fig. 5).

SignalP analysis revealed considerable variation in the predicted signal peptides among isolates for all three genes. Several clinical isolates showed high signal peptide probability values that were comparable to the reference strain, while others showed moderate or low probabilities. For certain isolates, no signal peptide was predicted. The distribution of SignalP scores is summarized in Table 3. The amino acid alignment generated from the translated sequences also displayed variability at the N-terminal region, which corresponded to the differences observed in the assembled 5′-end sequences.

**Table 3:** Predicted signal peptide probabilities for ACA1_187760, ACA1_252830, and ACA1_287530 derived from PCR-reassembled 5′-terminal sequences

Isolate	ACA1_187760		ACA1_252830		ACA1_287530	
	PCR	SignalP	PCR	SignalP	PCR	SignalP
ATCC_30010	+	0.9987	+	0.9998	+	0.9996
ATCC_50370	+	0.0031	‒	N/A	+	0
ATCC_50492	+	0.0609	+	0.1825	+	0.9998
NCKH_A	+	0.0774	+	0.9997	+	0.9952
NCKH_B	+	0.7922	+	0.8193	+	0.7678
NCKH_C	+	0.9471	+	0.0001	+	0.144
NCKH_D	+	0.9997	+	0	+	0.9082
NCKH_H	+	0.9995	+	0.0001	+	0.0003

These results confirm that the 5′ ends of these three lectins display substantial sequence diversity among clinical isolates at both the nucleotide and amino acid levels, and that this variation is reflected in the predicted signal peptide regions.

## Discussion

In this study, the three cyst wall lectins (ACA1_187760, ACA1_252830, and ACA1_287530) that formed clinical clusters showed marked diversity at their 5′ terminal sequences across *Acanthamoeba* isolates. Variation at the 5′ end is particularly relevant because this region typically contains the signal peptide that directs lectins to their appropriate positions within the cyst wall. Any alteration in this region has the potential to influence the final localization or function of the encoded proteins. Previous investigations of encystation have demonstrated that cyst wall assembly is a highly ordered process in which different lectins appear at specific stages and occupy distinct structural domains. These spatial and temporal patterns highlight the functional specialization of the lectin families during wall biogenesis. All lectins identified in our clinically associated clusters belong to the Luke families, which contribute to the endocyst layer and ostioles. These structures form the protective architecture of the cyst wall and participate in environmental sensing and excystation, since the operculum covering each ostiole is removed when the trophozoite emerges [12, 18, 29]. Given this biological context, the considerable 5′ sequence variation identified in our study raises the possibility that different isolates may produce lectins with altered N-terminal properties, which could modify how these proteins participate in cyst wall assembly. At present, it remains unclear whether the observed variation reflects true differences among isolates or limitations of currently available genome assemblies. Additional functional and structural studies will be required to determine how variation in cyst wall lectins influences cyst wall organization and the biology of encystation and excystation.

A multiomics analysis by Clément Bernard et al. in 2022 revisited the same set of 31 lectins during the early phase of encystation [30]. Among these genes, 23 were detected at the transcript level, and 16 were significantly differentially expressed. However, only six lectins were identified in the proteome, including one Jonah, four Luke, and one Leo, and none exhibited significant protein-level regulation under their experimental conditions. The authors proposed that this discrepancy may reflect differences in timing, since peak expression of many lectins may occur later than the 8-h window examined in their study.

Notably, the lectins identified in our work as forming clinically associated clusters were not among those previously detected by fluorescent tagging or proteomic profiling. Two interpretations remain possible. These lectins may still participate in encystation or excystation, but are expressed at stages that have not yet been captured by existing experimental designs. Alternatively, they may fulfill biological functions unrelated to cyst wall formation. Further temporal and functional studies will be required to clarify their roles.

This study did not assess the expression levels of lectins with 5′-end variation, and determining their transcriptional activity will be an important direction for future research. Evaluating gene expression in *Acanthamoeba* requires careful attention to potential artifacts from prolonged axenic cultivation, which can alter transcriptional profiles [31–33]. Moreover, the substantial genomic diversity among isolates makes it challenging to design universal primers for quantitative PCR (qPCR) or other quantitative assays. These considerations will be essential when investigating whether lectins with variable 5′ regions are expressed in clinical isolates and how their expression relates to cyst wall formation.

Although the expression status of these lectins remains unknown, the presence of marked 5′-end variation suggests that the cyst wall architecture of clinical isolates may differ significantly from that of ATCC_30010. Such differences may arise either from altered N-terminal features in expressed lectins or from the absence of lectins that are not expressed. Further functional studies will be necessary to determine how variation in cyst wall lectins contributes to structural diversity among isolates and whether these differences influence pathogenic potential.

## Conclusions

In this study, we examined cyst wall lectin genes across diverse *Acanthamoeba* isolates and observed notable sequence diversity associated with clinical clusters. Among the 31 previously described lectins, 8 showed clustering patterns enriched for clinical isolates, and three of these exhibited clear variation at their 5′ regions. Reconstruction of upstream sequences and signal peptide prediction based on PCR-confirmed clinical isolates further demonstrated diversity in the N-terminal regions of these lectins.

The biological implications of this variation remain uncertain, and additional work is needed to determine whether these sequence differences influence lectin expression, localization, or cyst wall assembly. Nevertheless, the results highlight cyst wall lectins as potential subjects for further investigation into isolate-level diversity in *Acanthamoeba*. Improved genome assemblies and stage-specific expression analyses will be important for future work. These approaches may help clarify whether sequence variation has any relationship to encystation biology or the behavior of clinical isolates. This study provides a preliminary framework for future functional studies aimed at understanding the diversity of cyst wall-associated genes among *Acanthamoeba* isolates.

## Data Availability

All data supporting the findings of this study are available within the paper.
